# Dendroclimatic response of *Pinus tabuliformis* Carr. along an altitudinal gradient in the warm temperate region of China

**DOI:** 10.3389/fpls.2023.1147229

**Published:** 2023-03-30

**Authors:** Peng Ning, Min Zhang, Tianyu Bai, Bin Zhang, Liu Yang, Shangni Dang, Xiaohu Yang, Runmei Gao

**Affiliations:** ^1^ College of Forestry, Shanxi Agricultural University, Taigu, Shanxi, China; ^2^ Zhongtiaoshan Forestry Bureau of Shanxi Province, Houma, Shanxi, China

**Keywords:** climate change, radial growth, altitude gradient, *Pinus tabuliformis* Carr., Zhongtiao Mountains

## Abstract

**Introduction:**

Global climate change can affect the sensitivity of tree radial growth to climate factors, but the specific responses of tree radial growth to microclimate along the altitudinal gradient in the long term are still unclear.

**Methods:**

In this study, the tree-ring width chronologies of *Pinus tabuliformis* Carr. in Shanxi Province of China were studied at three altitude gradients (1200-1300 m (low altitude), 1300-1400 m (medium altitude) and 1400-1500 m (high altitude)) during 1958-2017.

**Results:**

The results showed that (1) the climate background could be divided into two periods based on the Mann-Kendall test analysis: 1958–1996 was a stable period (mean annual temperature (MAT)=10.25°C, mean annual precipitation (MAP)=614.39 mm), and 1997–2017 was a rapid change period (MAT=10.91°C, MAP=564.70 mm), indicating a warming and drying trend in the study region. (2) The radial growth of *P. tabuliformis* at different altitudes showed inconsistent variation patterns. The tree radial growth at low and medium altitudes (CV=27.01% for low altitude and CV=24.69% for medium altitude) showed larger variation amplitudes during the rapid change period than that in the stable period (CV=12.40% for low altitude and CV=18.42% for medium altitude). In contrast to the increasing trend, the tree radial growth rates at the high altitude showed a decreasing trend across years. (3) In the stable period, the radial growth of *P. tabuliformis* at the low altitude showed a significantly negative response to temperature and a positive response to precipitation in May and June. The tree radial growth at the medium altitude was positively related to precipitation in June and minimum temperature in February. The tree growth at the high altitude was mainly positively correlated with the temperature in May and August. In the rapid change period, the radial growth of *P. tabuliformis* at the low altitude was affected by more meteorological factors than that in the stable period. Medium-altitude trees were positively influenced by precipitation in June and minimum temperature in January, whereas high-altitude trees responded positively to wind speed in February. (4) Along altitudinal gradients, tree radial growth was more related to temperature than precipitation in the stable period. The tree radial growth at the high altitude during the rapid change period was only affected by wind speed in February, whereas the tree radial growth at low and medium altitudes was mainly affected by temperature to a similar extent during the two periods.

**Discussion:**

The study indicated that tree growth-climate response models could help deeply understand the impact of climate change on tree growth adaptation and would be beneficial for developing sustainable management policies for forest ecosystems in the transition zone from warm-temperate to subtropical climates.

## 1 Introduction

The global warming trend continues (WMO, 2022), and forests will be greatly affected (Bonan, 2008). However, knowledge about how forests have responded to continuous warming since the latter half of the last century has not been well documented (Matías et al., 2017). Therefore, exploring the impact of climate change on forest ecosystems has become an important scientific issue (Babst et al., 2018). With accurate dating, high resolution, long time series and retention of rich tree growth information (Fritts, 1976), tree rings have been widely used by scholars from various countries for paleoclimate reconstruction, assessing the response of tree growth to climate change, and revealing the impact of temperature increase on regional vegetation (Zhang and Qiu, 2007; Liang et al., 2009; Fan et al., 2010; Wang et al., 2017).

Climate warming since the latter half of the 20^th^ century has changed the environmental conditions and growth trends of trees (Rai et al., 2020), as well as the way trees interact with the climate (Babst et al., 2019). It also alters tree physiological processes and ecosystem productivity in an interactive and complex manner (Keenan et al., 2013; Reed et al., 2018). Studies have shown that the threshold effect leads to changes in the relationship between tree radial growth and climate (Jacoby and D’Arrigo, 1995). For example, under the influence of climate change, the changes in tree-ring width showed synchronization with climate factor fluctuations (Shestakova et al., 2016); however, temperature increases caused growth declines in some tropical forests (Feeley et al., 2007). This showed that trees in different climatic zones respond differently to climate change (Zhou et al., 2021). Altitudinal differences also increase the complexity of the response of tree growth to climate change (Zhang et al., 2022).

The altitude gradients are equivalent to natural cooling and humidifying platforms. With increasing altitude, the relationship between tree growth processes and climate factors changes (Salzer et al., 2014). Trees at different altitudes can adopt different ecological strategies to respond wisely to vertical zonal changes in mountain forest climates (Zhang et al., 2022). It is generally believed that the radial growth of trees at high altitudes is mainly limited by temperature, and at low altitudes, it is mainly related to precipitation (Wu et al., 2017). However, with global warming, the limiting effect of temperature on tree growth at high latitudes is gradually diminishing. The effect of precipitation on tree growth is becoming stronger (Büntgen et al., 2008; Loehle, 2009). This may be because there are different response mechanisms of tree radial growth to temperature and precipitation along altitudinal gradients. Therefore, it is important to explore how tree redial growth responds to climate change at different altitudes in the same region.

Currently, under the context of a warming and drying climate, the Qinling Mountains, an important geographic and climate dividing line in China, have attracted the interest of many scholars for their climate change and possible impacts (Li et al., 2022). Similarly, the Zhongtiao Mountains, which belong to the same warm-temperate to subtropical climate transition zone as the Qinling Mountains, are also a sensitive and vulnerable area for tree growth in response to climate change. In addition, *P. tabuliformis* is an important timber-tree species in North, Northwest and Northeast China, with a wide natural distribution area, which has a prominent strategic position in maintaining regional ecological security and significant economic advantage. Moreover, it is one of the dominant tree species in the Zhongtiao Mountains and the main forestation species in this area. Considering that tree rings have the characteristics of climatic sensitivity, accurate dating, strong continuity and high resolution, they have been widely used in dendroclimatological studies. For example, the tree rings of *P. tabuliformis* can be used to predict climate trends based on data about January-June precipitation changes over the last 296 years (1724-2019) (Cao et al., 2021) and the annual precipitation since 1686 in Ningwu County of China’s Shanxi Province (Li et al., 2006). However, the relationship between tree rings and micrometeorological factors at different altitudes is unclear. Therefore, it is necessary to explore the radial growth and dynamic response patterns of *P. tabuliformis* at different altitude gradients under climate change using the tree-ring method.

The aim of this study was to (1) investigate the pattern of radial growth of *P. tabuliformis* in response to climate factors at different altitude gradients; (2) quantify the contribution of climate factors to the radial growth of *P. tabuliformis* at different altitudes; and (3) reveal the radial growth trend of *P. tabuliformis* under the warming climate in the Zhongtiao Mountains. The study may provide sustainable management for forests with climate change in the Zhongtiao Mountains of China’s Shanxi Province.

## 2 Materials and methods

### 2.1 Study sites

**Figure 1:**
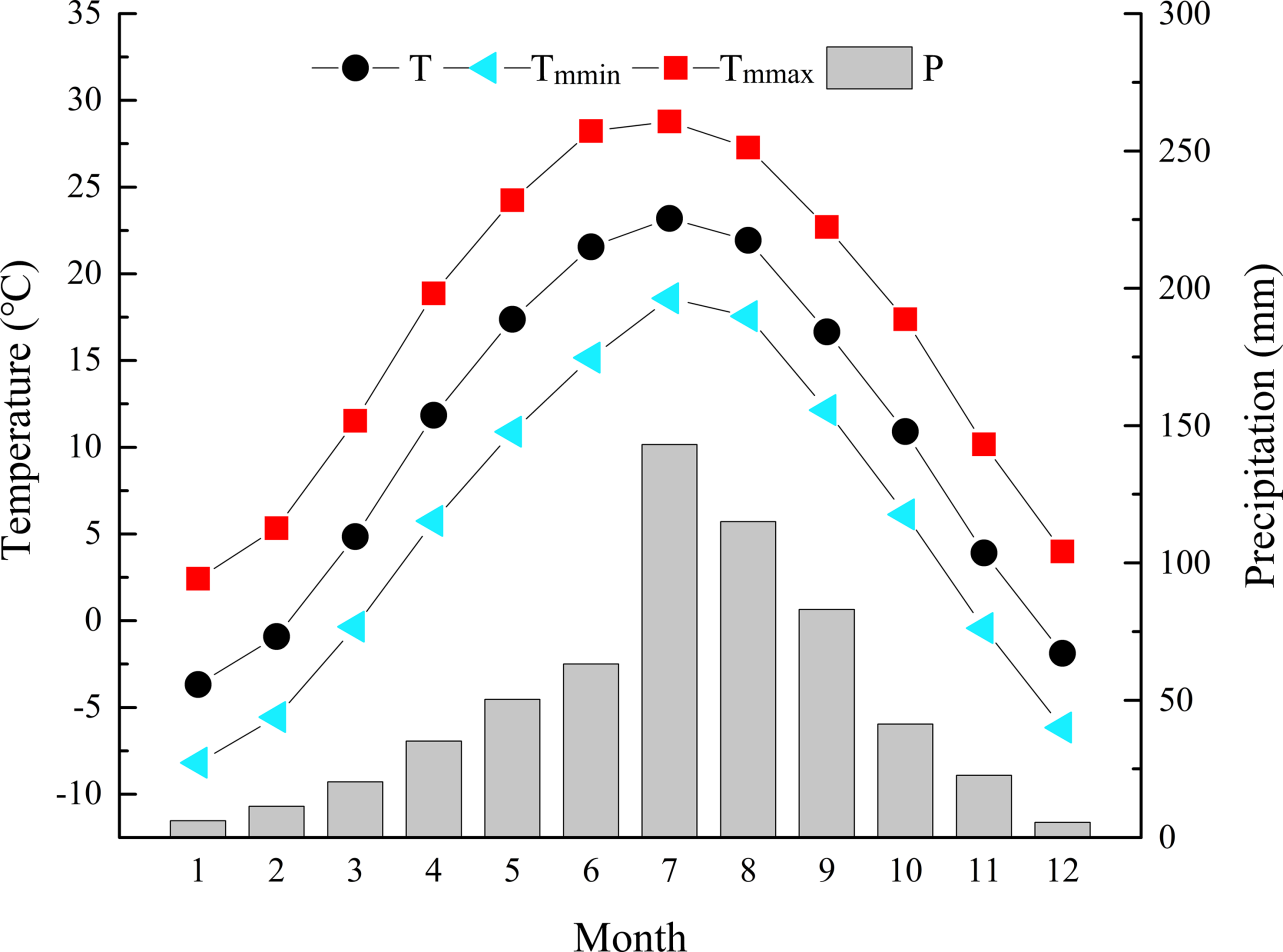
Climate records of monthly mean temperature (T), monthly mean minimum temperature (T_mmin_), monthly mean maximum temperature (T_mmax_), and monthly precipitation (P) during 1958-2017 at the Qinshui County Meteorological Station.

Zhongtiao Mountain (34°36´-35°53´N and 115°15´-112°37´E) is located in southern Shanxi Province. It is a long, narrow, northeast−southwest trending mountain system, with the southern remnants of the Taihang Mountains on the east and the Yellow River cutting it off from the Huashan Mountains on the west. Lying in the transition zone from warm-temperate to subtropical climate zones, it belongs to a warm temperate continental monsoon climate. It is hot and rainy in summer and cold and dry in winter. According to the meteorological data of the nearest Qinshui County National Weather Station (35°41′ N, 112°11′ E), the mean annual temperature (MAT) was 3-11°C, with the coldest in January (-8.2°C) and the hottest in July (28.8°C). The mean annual precipitation (MAP) was 600-720 mm, mainly concentrated in June, July, August and September, accounting for 69.67% of the annual precipitation (**Figure 1**).

### 2.2 Tree-ring data and chronologies

In 2018, based on the distribution range of *P. tabuliformis* in the Zhongtiao Mountains, we selected 18 mature dominant trees as standard trees at low altitude (L, 1200-1300 m, n=7), medium altitude (M, 1300-1400 m, n=5) and high altitude (H, 1400-1500 m, n=6). The north−south direction and the location at breast height (1.3 m) were marked on the tree trunks. After felling, disks approximately 3-5 cm thick were intercepted at breast height (1.3 m) and marked with the standard tree number, north−south direction, etc. In the laboratory, the nonmarked surfaces were sanded and polished until the ring boundary of the disks was clearly visible. The disks were scanned by MICROTEK-3200A3L. Then, the ring width was measured using WinDENDEO (accuracy 0.001 mm) in eight directions. That is, we defined the due south direction as 0 degrees and recorded one core every 45 degrees in a clockwise direction, with a total of eight cores recorded for each disk. Meanwhile, we determined the accurate calendar age of each ring following a cross-dating procedure using the “dplR” package (Bunn, 2008) in R software (R Development Core Team, 2020). The age of each ring with dating errors was remeasured and cross-dated again. The unqualified cores that could not be dated (such as the severely damaged samples) were removed. Finally, 106 cores were used in this study.

The growth of trees is not only influenced by environmental factors but also controlled by their own physiology. To reduce the impact of individual differences between trees, the raw tree-ring width sequence was transformed into a standard and dimensionless ring-width index using the function “detrend” from the “dplR” package (Bunn, 2008) in R software. The standard chronology can accurately reflect the changes in tree growth patterns and the associated climatic factors (Wang and Yang, 2021). Additionally, the detrended tree-ring width indices were averaged using a robust biweight mean, with the function “chron” from the “dplR” package in R, to obtain the standard chronology. The quality of the chronologies was evaluated by several statistical parameters (**Table 1**). Specifically, mean sensitivity (MS) effectively reflects the interannual variability of the width between successive rings. For example, a high MS value indicates that the ring-width variation is more sensitive to climate than a low MS value. The signal-to-noise ratio (SNR) and expressed population signal (EPS) indicate the representativeness of the sample, with larger values indicating more environmental information for analysis in the chronology. For example, an EPS value above 0.85 generally indicates a qualified and reliable chronology (Wigley et al., 1984). The first-order autocorrelation (AC1) was generally used to identify whether the tree-ring width of the current year has a relationship with the climate factors of the previous year.

**Table 1:** Statistical parameters of the standardized chronology of *P. tabuliformis* at different altitude gradients.

Chronology indicator	Altitudes		
	Low	Medium	High
Elevational range (m) 0)	1200-1300	1300-1400	1400-1500
Number of samples	43	32	31
First-order autocorrelation (AC1)	0.559	0.826	0.452
Mean sensitivity 1 (MS1)	0.356	0.262	0.288
Mean sensitivity 2 (MS2)	0.349	0.256	0.286
Signal-to-noise ratio (SNR)	6.068	7.499	5.972
Expressed population signal (EPS)	0.859	0.882	0.857

### 2.3 Meteorological data

In this study, the daily meteorological data during 1958-2017 observed in the Qinshui County National Weather Station were used, as it is close to the study site and the data could represent the climate background in the study area. After calculation, we obtained climate indices, including monthly precipitation (P, mm), monthly temperature (i.e., mean temperature (T), maximum temperature (T_max_), minimum temperature (T_min_), mean maximum temperature (T_mmax_), mean minimum temperature (T_mmin_), °C), monthly relative humidity (RH, %), monthly sunshine hours (S, h), monthly average wind speed (WS, m/s), and standardized precipitation-evapotranspiration index (SPEI). Specifically, SPEI was calculated by the difference between standardized precipitation and potential evapotranspiration using the “SPEI” package (Beguería and Vicente-Serrano, 2009) in R software. For example, a negative SPEI value indicates a lack of water, which may depress tree growth. Conversely, a positive SPEI value indicates that tree growth can be promoted (Vicente-Serrano et al., 2010). Considering the “lag effect” of climate factors (Fritts, 1976), the climate data from the previous June to the current September during the whole period of 1958–2017 were used to investigate the effects of intra-annual climate factors on tree radial growth.

### 2.4 Data analyses

The Mann-Kendall test (Gocic and Trajkovic, 2013) was used to analyze the trend of meteorological data during 1958-2017. The correlation coefficient (r) of standard chronologies at different altitudes with monthly climate variables was calculated using the “treeclim” package (Bunn, 2008) in R software. Moreover, according to previous studies (Dormann et al., 2013; Harrison et al., 2018), |r| < 0.7 is an appropriate indicator that the tree’s radial growth at different altitudes is independent. To quantify the effects of climatic factors on tree radial growth, multivariate stepwise regression analysis was performed by the “step” function from the R package “stats” (Zhang et al., 2020) using the tree-ring width index as the predictor variable and climatic factors as explanatory variables to obtain the optimal regression model. Before regression, the values of climatic factors were standardized. To quantify the interpretation rate of tree radial growth by climatic factors, the *R* ^2^ of each explanatory variable in the regression model was used as the explanatory rate (the sum of the explanatory rates of each factor was the total *R* ^2^ of the regression model, which could actually reflect the explanatory amount of each factor for tree radial growth), and the percentage of the absolute value of the standardized regression coefficient of each explanatory variable to the sum of the absolute values of the regression coefficients of all explanatory variables was used as the contribution rate of climatic factors to tree radial growth (Le Bagousse-Pinguet et al., 2019). Data analysis was performed in R language, and the significance level was α=0.05.

## 3 Results

### 3.1 Climatic trends

**Figure 2:**
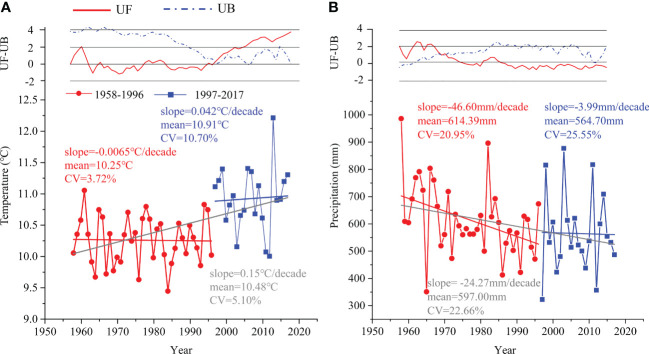
The variation and abrupt change in **(A)** mean annual temperature and **(B)** mean annual precipitation during 1958–1996 (red circle) and 1997–2017 (blue square) based on the Mann-Kendall test. UF: sequence of forward test values, UB: sequence of reverse test values.

According to the results of the Mann-Kendall test analysis of the meteorological data during 1958-2017 in the study area, the mean annual temperature (MAT) increased at a rate of approximately 0.15°C/decade, whereas the mean annual precipitation (MAP) decreased at a rate of 24.27 mm/decade (**Figure 2**). Additionally, the UF and UB curves of MAT and MAP had crossing points in 1996 and 1969, respectively (**Figure 2**). We considered 1996 to be an abrupt point in climate change because the UF value of MAT before 1996 was almost less than zero, and 1969 was the initial stage of the study.

There was a decreasing trend for MAP in both periods (i.e., 1958-1996 and 1997-2017), which was similar to the MAP of the whole period (**Figure 2B**). Conversely, there were increasing trends for MAT in the period of 1997-2017 and the whole time, except for 1958-1996 (**Figure 2A**). Additionally, the MAT of 1997-2017 had a larger coefficient of variation (CV=10.70%) than that of 1958-1996 (CV=3.72%). Collectively, we divided the entire study period into relatively stable (1958-1996) and rapid change periods (1997-2017).

### 3.2 Chronology characteristics and tree radial growth along altitudinal gradients

In this study, the statistical parameters of the standard chronology had generally similar variation trends at three different altitude gradients (**Table 1**). For example, the values of first-order autocorrelation (0.826), signal-to-noise ratio (7.499) and expressed population signal (0.882) were the highest at medium altitude, followed by low altitude (0.559, 6.068 and 0.859, respectively), and finally high altitude (0.452, 5.972 and 0.857, respectively).

**Figure 3:**
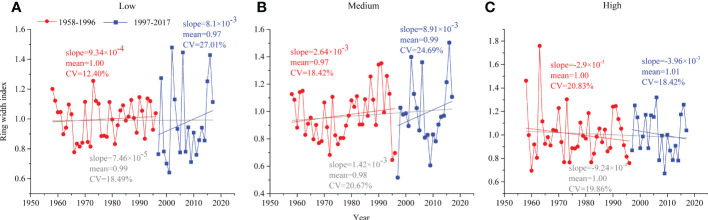
Tree radial growth of *P. tabuliformis* in natural forests during 1958–1996 (red circle) and 1997–2017 (blue square) at **(A)** low altitude, **(B)** medium altitude and **(C)** high altitude.

Comparing the characteristic curves of the ring width index (RWI) changes at different altitude gradients of the two periods, the RWI of low and medium altitudes increased slowly before 1996 (the slopes of the regression line were 9.34×10^-4^ and 2.64×10^-3^, respectively) and increased rapidly after 1996 (8.1×10^-3^ and 8.91×10^-3^, respectively), while the RWI of high altitude decreased at both periods (**Figure 3**).

### 3.3 Response of tree radial growth to climate at different altitude gradients

**Figure 4:**
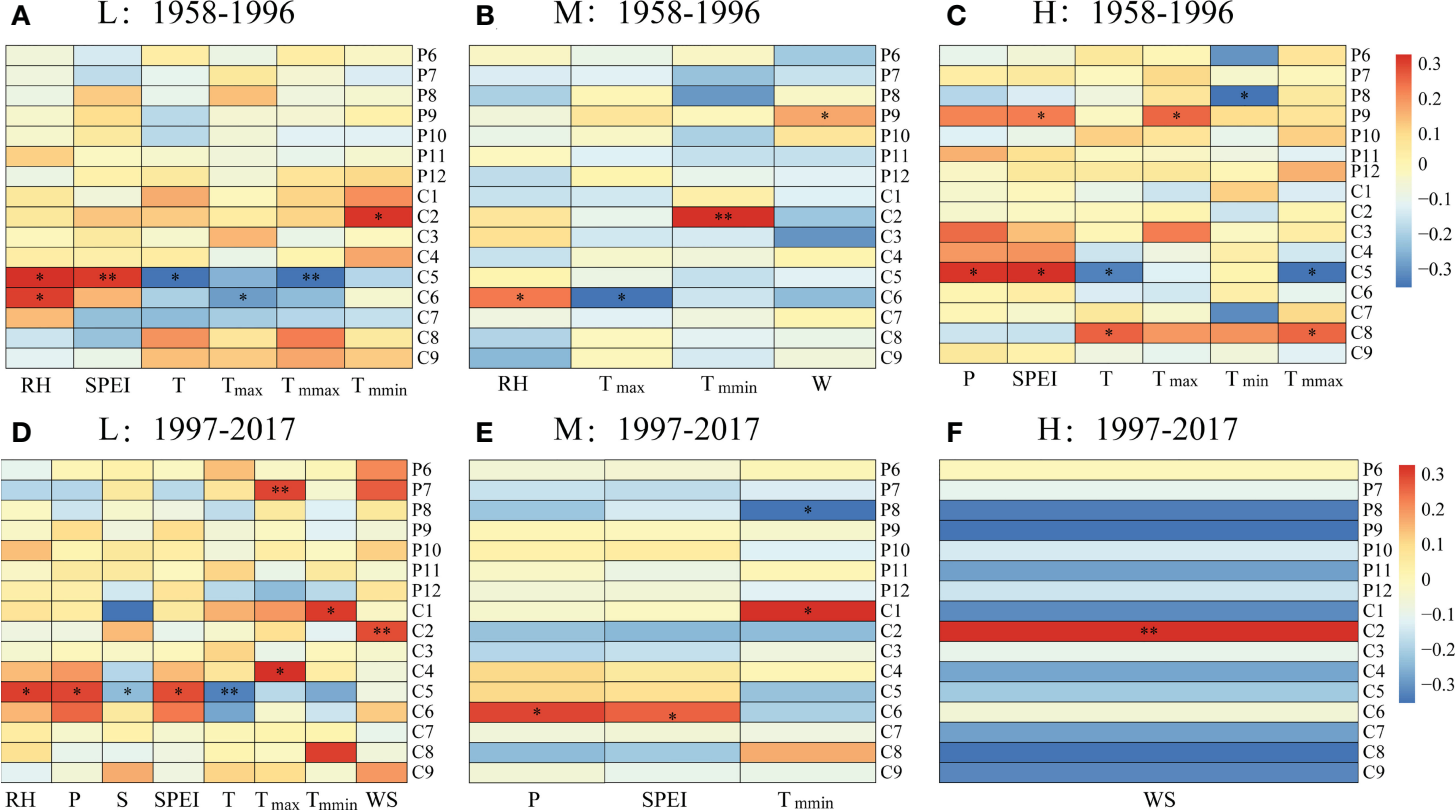
Correlations between the ring width index (RWI) and monthly climatic factors at **(A, D)** low (L), **(B, E)** medium (M) and **(C, F)** high (H) altitudes during **(A–C)** 1958–1996 and **(D–F)** 1997–2017. *, *p* < 0.05; **, *p* < 0.01. RH, relative humidity; T, monthly mean temperature; P, monthly precipitation; SPEI, standardized precipitation-evapotranspiration index; WS, monthly mean wind speed; S, monthly mean sunshine hours; T_max_, monthly maximum temperature; T_mmax_, monthly mean maximum temperature; T_min_, monthly minimum temperature; T_mmin_, monthly mean minimum temperature. For the labels on the right, P indicates the previous year; C indicates the current year, and the numbers indicate the month.

For the period 1958-1996, the tree radial growth of *P. tabuliformis* at the low altitude had a significantly negative correlation with monthly mean temperature in May (T_C5_, *p* < 0.05), maximum temperature in June (T_max-C6_, *p* < 0.05) and mean maximum temperature in May (T_mmax-C5_, *p* < 0.01) but had a significantly positive correlation with monthly relative humidity in May (RH_C5_, *p* < 0.05) and June (RH_C6_, *p* < 0.05), as well as the SPEI in May (SPEI_C5_, *p* < 0.01) (**Figure 4A**). Consistent with low altitude, the tree radial growth at medium altitude had a significantly negative correlation with T_max-C6_ (*p* < 0.05) and a positive correlation with RH_C6_ (*p* < 0.05) (**Figure 4B**). However, the radial growth at high altitude had a significantly negative correlation with T_C5_ (*p* < 0.05), T_mmax-C5_ (*p* < 0.05) and monthly minimum temperature in August of the previous year (T_min-P8_, *p* < 0.05) but had a significant positive correlation with T_C8_ (*p* < 0.01), T_mmax-C8_ (*p* < 0.01), monthly maximum temperature in September of the previous year (T_max-P9_, *p* < 0.01), monthly mean precipitation in May (P_C5_, *p* < 0.05), as well as SPEI in May of the current year (SPEI_C5_) and September of the previous year (SPEI_P9_, *p* < 0.05) (**Figure 4C**).

Compared with the period of 1958-1996, the radial growth of *P. tabuliformis* at different altitudes showed more complex relationships with climatic factors during 1997-2017 (**Figure 4**). In the low-altitude zone, radial growth had a significantly negative correlation with monthly mean sunshine hours (S_C5_, *p* < 0.05) and T_C5_ (*p* < 0.01) but a positive correlation with RH_C5_ (*p* < 0.05), P_-5_ (*p* < 0.05), SPEI_C5_ (*p* < 0.05), T_max-C5_ (*p* < 0.05), T_max-P7_ (*p* < 0.01), T_mmin-C1_ (*p* < 0.05) and wind speed in February of the current year (WD_C2_, *p* < 0.01).

Unlike at the low altitude, the radial growth at medium and high altitudes was significantly correlated with only a few meteorological factors (**Figure 4**). For example, the radial growth at medium altitudes had a significantly positive correlation with P_-6_, SPEI_C6_ and T_mmin-C6_ but a negative correlation with T_mmin-P8_ (*p* < 0.05, **Figure 4E**). Additionally, the radial growth at the high altitude only showed a significantly positive correlation with WS_C2_ (*p* < 0.01, **Figure 4F**).

### 3.4 Contribution of climatic factors to the radial growth of trees

**Figure 5:**
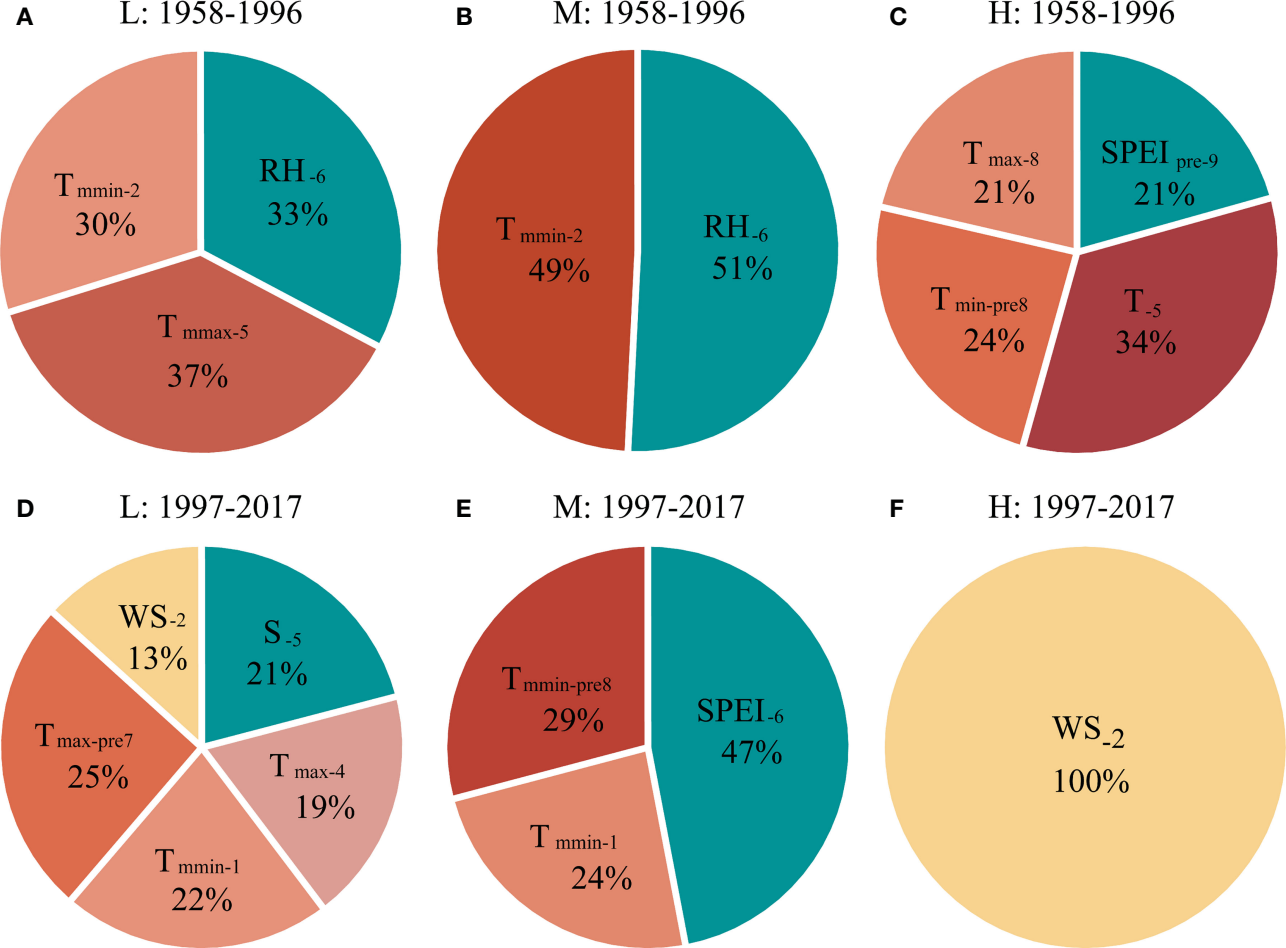
The contribution of different climatic factors in explaining the tree radial growth of *P. tabuliformis* at **(A, D)** low (L), **(B, E)** medium (M) and **(C, F)** high (H) altitudes during **(A–C)** 1958–1996 and **(D–F)** 1997–2017, respectively, based on the optimized regression model (see **Table 2**). See **Figure 4** for abbreviations of meteorological factors.

The results of multivariate stepwise regression analysis showed that the influence of temperature and precipitation on the radial growth of *P. tabuliformis* varied with altitude (**Table 2**). Moreover, the contributions of temperature and precipitation to the radial growth of *P. tabuliformis* were different (**Figure 5**).

**Table 2:** Estimates of the multivariate stepwise regression model of the effect of climate factors on the radial growth of *P. tabuliformis.*.

Time period	Altitude	The multivariate stepwise regression model	
1958-1996	L	RWI=0.998+0.037RH_C6_-0.043T_mmax-C5_+0.034T_mmin-C2_	*R* ^2 =^ 0.386, *p*<0.01
	M	RWI=0.972+0.073RH_C6_+0.070T_mmin-C2_	*R* ^2 =^ 0.358, *p*<0.01
	H	RWI=0.990+0.056SPEI_P9_-0.091T_C5_-0.066T_min-P8_+0.058T_max-C8_	*R* ^2 =^ 0.478, *p*<0.01
1997-2017	L	RWI=0.977-0.094S_C5_+0.084T_max-C4_+0.097T_mmin-C1_+0.012T_max-P7_+0.060 WS_C2_	*R* ^2 =^ 0.858, *p*<0.01
	M	RWI=0.997+0.129SPEI_C6_+0.065T_mmin-C1_-0.079T_mmin-P8_	*R* ^2 =^ 0.695, *p*<0.01
	H	RWI=1.005+0.132WS_C2_	*R* ^2 =^ 0.511, *p*<0.01

RWI, the ring width index of standard chronology; RH_C6_, the relative humidity in June of current year; T_mmax-C5_, the monthly mean maximum temperature in May of current year; T_mmin-C2_, T_mmin-C1_, the monthly mean minimum temperature in February and January, respectively of current year; SPEI_P9_, the standardized precipitation-evapotranspiration index (SPEI) in September of previous year; T_C5_, the monthly mean temperature in May of current year; T_min-P8_, the monthly minimum temperature in August of previous year; T_max-C8_, T_max-C4_, the monthly maximum temperature in August and April, respectively of current year; S_C8_, the monthly sunshine hours in August of current year; T_max-P7_, the monthly maximum temperature in July of previous year; WS_C2_, the monthly mean wind speed in February of current year; SPEI_C6_, the monthly SPEI in June of current year; T_mmin-P8_, the monthly mean minimum temperature in August of previous year.

During the period of 1958-1996, the current year’s temperature explained 67% (37% for T_mmax-C5_ and 30% for T_mmin-C2_), and precipitation explained 33% (RH_C6_) of the variation in radial growth of *P. tabuliformis* at low altitude, respectively (**Figure 5A**). For radial growth at the medium altitude, the contributions of temperature (T_mmin-C2_) and precipitation (RH_C6_) in the current year were 49% and 51%, respectively (**Figure 5B**). At the high altitude, the temperature of the previous and current years explained 79% (34% for T_C5_, 21% for T_max-C8_ and 24% for T_min-P8_), and the precipitation of the previous year explained 21% (SPEI_P9_) of the radial growth variation in *P. tabuliformis* (**Figure 5C**).

During the period of 1997-2017, the contribution rate of temperature to the radial growth variation of *P. tabuliformis* at the low altitude was 66% (25% for T_max-P7_, 22% for T_mmin-C1_ and 19% for T_max-C4_), and that of wind speed (WS_C2_) and sunshine hours (S_C5_) was 13% and 21%, respectively (**Figure 5D**). At the medium altitude, the contribution rates of temperature and precipitation to the variation in radial growth of *P. tabuliformis* were 53% (29% for T_mmin-P8_ and 24% for T_mmin-C1_) and 47% (SPEI_C7_), respectively (**Figure 5E**). At the high altitude, the radial growth variation of *P. tabuliformis* was only significantly affected by the wind speed in February of the current year (**Figure 5F**).

## 4 Discussion

### 4.1 Chronological characteristics and change patterns at different altitudes

In this study, most of the statistical parameters (e.g., SNR, EPS and AC1) of the standard chronology at the medium altitude exhibited higher values than those at low and high altitudes, but the value of mean sensitivity had the opposite trend (**Table 1**), which was consistent with the findings of previous studies (Fritts, 1976; Shrestha et al., 2017; Panthi et al., 2020). These results indicated that compared with that at low and high altitudes, the tree-ring width at the medium altitude exhibited lower sensitivity to climate change. This may be because the climatic conditions at medium altitudes were suitable, and other topographic or microenvironmental factors had an impact on tree growth (Weemstra et al., 2021).

Previous studies were conducted at sites in the arid zone, where spring precipitation may be the limiting factor for tree growth (Gou et al., 2004). However, our study area is in the transition zone from a warm temperate to subtropical climate zone, with relatively abundant precipitation, indicating that the growth of trees may be less affected by precipitation than other disturbance events. For example, our study area belongs to a warm temperature continental monsoonal climate zone, with cold and dry winters and windy springs. A previous study showed that the average daily wind speed in winter (pre-November to January) was approximately 3 m/s, which can directly cause the trunk and branches of the tree to break; furthermore, the wind also caused snow to accumulate, indirectly causing the breakage of the trunks and branches (Firm et al., 2009). Additionally, physiological drought caused by strong winds may also be a main cause affecting tree growth (Yu et al., 2016). In the present study, the radial growth of *P. tabuliformis* at high altitudes was more vulnerable to climate change than that at low and medium altitudes (**Table 1**), which was inconsistent with the previous findings by Zhang et al. (2015). This may be because the lowest elevation in Zhang et al.’s study was 2330 m, while the highest elevation in this study was 1500 m, indicating that tree radial growth at different altitudes was affected by microenvironmental factors.

### 4.2 Tree growth-climate response models at different altitudes

In this study, the radial growth of *P. tabuliformis* among the three altitudes had different degrees of variation and inconsistent relationships with climatic factors in the relatively stable (1958-1996) and rapid change periods (1997-2017) (**Figure 3**).

At the low altitude, the radial growth of *P. tabuliformis* was inhibited by the maximum temperature in May and June during the stable period (**Table 2**, **Figure 4A**), which was consistent with previous studies conducted in the eastern and northern Qinling Mountains (Shi et al., 2012; Sun and Liu, 2016). A study conducted in a lower montane site of the Swiss Plateau (805 m a.s.l.) showed that tree-ring growth had a significantly negative correlation with June temperature (Meyer and Braker, 2001). This may be because the higher temperature of the early growing season accelerated soil moisture loss, and tree roots could not obtain the water required for photosynthesis, leading to tree radial growth restriction (Peng et al., 2011). Furthermore, the radial growth of *P. tabuliformis* was enhanced by the relative humidity in May and June during the stable period (**Table 2**, **Figure 4A**). This is presumably because May and June are early in the growing season, when the water requirement for rapid shoot growth and dry matter accumulation increases (Yang et al., 2004). Meanwhile, the increase in relative humidity of the early growing season helps to replenish the water required for the physiological activities inside the trees and accelerates cell division, which leads to an increase in photosynthetic products and promotes the growth of the tree (Cui et al., 2021). Additionally, we found that tree radial growth at low altitudes was enhanced by the maximum temperature in April during the rapid change period (**Table 2**, **Figure 4D**). This phenomenon was also previously found in other regions of China (Yang et al., 2022). This is likely because the moderate warming in April was useful to break dormancy, promote snow melting and increase soil moisture, indirectly enhancing tree growth (Dang et al., 2007).

For the medium altitude, the radial growth of *P. tabuliformis* showed a significantly positive correlation with minimum temperature in February in the stable period (**Table 2**, **Figure 4B**). This was consistent with previous findings on the southern slope of the Qinling Mountains (Liu and Shao, 2003). The reason may be that February is the dormant period of plants, when the minimum temperature can affect the mechanical damage of trees and the reserve of tree water requirements (Liu et al., 2009). Conversely, the relationship between radial growth and the maximum temperature in June during the stable period was significantly negative (**Figure 4B**). For example, the increase in temperature of the growing season will result in the closure of stomata to reduce transpiration, thereby decreasing the photosynthetic rate and influencing organic matter synthesis and storage, which is detrimental to the growth of trees in the current year and even affects the growth of trees in the following year (Chen et al., 2011). In addition, the elevated summer temperature tended to induce drought events, which caused the embolism of xylem conduits and inhibited tree growth (Silva and Anand, 2013; Subedi and Sharma, 2013; Deslauriers et al., 2014). However, in the rapid change period, tree radial growth had a significant and negative correlation with the minimum temperature in August of the previous year (**Figure 4E**). A previous study showed that higher temperatures in the growing season caused stronger evapotranspiration, leading to soil water loss and stomatal closure, which in turn affected plant photosynthesis and inhibited the development of cells in the cambium (Tian et al., 2017).

Relative to the low and medium altitudes, tree radial growth at the high altitude during the stable period was affected by the temperature and precipitation of the current year as well as in the previous year (**Figure 4C**). For example, there was a significantly positive correlation between tree radial growth and the mean and maximum temperatures in August of the current year (**Figure 4C**). This may be related to the fact that higher temperatures can effectively prolong the length of the growing season in the high-altitude zone (Vitasse et al., 2018). Similarly, a previous study conducted at high altitudes in the Changbai Mountains showed that the higher temperature of the growing season was beneficial to the radial growth of trees (Wang et al., 2013). Moreover, there was a significantly positive correlation between tree radial growth at high altitudes and precipitation and SPEI in May during the stable period (**Figure 4C**). This may be because more precipitation in the early growing season helps to alleviate soil moisture deficits, increase the photosynthetic rate of trees, and facilitate carbohydrate synthesis and accumulation, thus promoting tree growth (Deslauriers et al., 2016; Chen et al., 2017). However, tree radial growth at the high altitude during the rapid change period was only influenced by the wind speed in February of the current year (**Table 2**, **Figure 4F**). One possible explanation is that winter precipitation in the study area mostly occurs in the form of snowfall, and low-temperature winds from the Siberian continent in February help the snowfall serve as an insulation layer for tree roots against frostbite (Reinmann and Templer, 2016).

### 4.3 Effect of warm and dry climate on the growth of *P. tabuliformis*

In our study, meteorological data recording showed an increase in temperature (from 10.25°C to 10.91°C) and a decrease in precipitation (from 614.39 mm to 564.70 mm) from 1958-1996 to 1997-2017 (**Figure 2**), leading to a trend toward a warmer and drier climate.

At the low altitude, the radial growth of *P. tabuliformis* in warm and dry climates (i.e., 1997-2017) had slightly lower mean values (0.97 vs. 1.00) and a higher coefficient of variation (CV, 27.01% vs. 12.40%) compared to the preclimate change period (i.e., 1958-1996) (**Figure 3A**). A previous study showed that the 600 mm precipitation isoline distinguished the response of tree ring width to climate, and the temperature south of this isoline significantly affected tree growth (Liu et al., 2019). This may be one reason for explaining our result, considering the area of our study located south of the 600 mm precipitation isoline.

Similar to the low altitude, the radial growth of *P. tabuliformis* at medium altitude in the period of 1997-2017 had higher CV (24.69% vs. 18.42%) and slightly higher mean values (0.99 vs. 0.97) than that of 1958-2016 (**Figure 3B**). Most likely, appropriate increases in temperature, within the threshold range of the optimal growth temperature of trees, can promote the absorption of water and nutrients, which in turn stimulates tree growth (Körner, 2007). As our study showed, the contribution of temperature for low (66% vs. 67%) and medium (49% vs. 53%) altitudes was almost equal over the two periods (**Figure 5**).

In contrast to the findings at medium and low altitudes, the radial growth of *P. tabuliformis* at the high altitude tended to decrease in both periods (**Figure 3**). This may be because high-altitude *P. tabuliformis* grows mainly in sandy and rocky areas with poor water storage capacity, where trees are susceptible to drought stress due to soil moisture being depleted, thus inhibiting tree radial growth (Girardin et al., 2016). Therefore, the negative effects of drought stress caused by rising temperatures on tree growth at the high altitude may be greater than the positive effects of increased precipitation with increasing altitude (Babst et al., 2019).

## 5 Conclusions

In this study, according to the results of the Mann-Kendall test, the climate background was divided into two periods: a relatively stable period (1958-1996) and a rapid change period (1997-2017). Changes in temperature (from 10.25°C to 10.91°C) and precipitation (from 614.39 mm to 564.70 mm) over the two periods indicated that the climate became warmer and drier in this region.

The radial growth of *P. tabuliformis* along the altitudinal gradients responded differently to the changing climate. Compared with the tree radial growth during the relatively stable period, the tree radial growth at low and medium altitudes increased faster with increasing temperature during the rapid change period. Conversely, a decreasing trend in radial growth at high altitudes was found throughout the whole period.

Additionally, the relationship between tree radial growth and climatic factors varied with altitude and varied in different climatic periods. For the trees at the low altitude, tree radial growth in the rapid change period was affected by more meteorological factors than that in the relatively stable period, although the temperature was the main influencing factor in both periods. Similar phenomena could be found at medium altitudes. However, tree radial growth at high altitudes during the rapid change period was only affected by wind speed in February, despite the temperature explaining 79% of the variation in radial growth in the relatively stable period.

Therefore, this study indicated that climate response models for tree radial growth along altitudinal gradients will be useful for providing more accurate suggestions for the future implementation of forest management policies in *P. tabuliformis* plantations under the background of global warming. Furthermore, it is helpful to better understand the dynamic responses of forest ecosystems to climate change, as well as to predict the impact of future global change on terrestrial ecosystems.

## Data availability statement

The raw data supporting the conclusions of this article will be made available by the authors, without undue reservation.

## Author contributions

PN (First Author):Methodology, Software, Investigation, Formal Analysis, Writing-Original Draft; MZ: Investigation; TB: Investigation; BZ: Data Curation; LY: Investigation; SD: Writing, Editing; XY: Software; RG (Corresponding Author):Conceptualization, Supervision, Editing. All authors contributed to the article and approved the submitted version.
